# Valoración de la formación de niños en reanimación cardiopulmonar mediante cuentos y dibujos animados

**DOI:** 10.23938/ASSN.1061

**Published:** 2023-12-26

**Authors:** Manuel Pardo Ríos, Petronila Mireia Alcázar Artero, Carolina Cánovas Martínez, José Manuel Salas Rodríguez, Mercedes Cánovas Martínez, María Isabel Rodríguez Entrena, Ana Belén Ocampo Cervantes, Esther Chicharro Luna, Laura Juguera Rodríguez

**Affiliations:** 1 Universidad de Murcia UCAM Universidad Católica de Murcia Grupo de Investigación de Nuevas Tecnologías para la Salud Guadalupe Murcia Spain; 2 Gerencia de Urgencias y Emergencias Sanitarias 061. Región de Murcia Murcia Spain; 3 Hospital Clínico Universitario Virgen de la Arrixaca El Palmar Murcia Spain; 4 Consejería de Educación y Universidades de la Región de Murcia Murcia Spain; 5 Universidad de Murcia UCAM Universidad Católica de Murcia Facultad de Ciencias de la Salud Murcia Spain; 6 Universidad Miguel Hernández de Elche Universidad Miguel Hernández Departamento de Ciencias de Comportamiento y Salud Sant Joan Alicante Spain

**Keywords:** Formación, Reanimación Cardiopulmonar, Maestros, Familia, Niños salvan vidas, Cardiopulmonary Resuscitation, Education, School Teachers, Family, Kids Save Lives

## Abstract

**Fundamento:**

El objetivo de este estudio es comparar los resultados obtenidos en escolares formados en RCP por progenitores en el ambiente familiar y por profesores en el ambiente educativo.

**Método:**

Estudio aleatorizado del aprendizaje de RCP en escolares de Educación Primaria (1º y 2º curso) de la Región de Murcia. Los progenitores (grupo familia, GF) y profesores (grupo maestros, GM) han participado como formadores a través de un material didáctico adaptado para escolares (un cuento y un vídeo de dibujos animados) de la serie educativa *Jacinto y sus Amigos*©. Se evaluaron ocho conocimientos y cinco habilidades prácticas.

**Resultados:**

Se seleccionaron 160 escolares y terminaron el estudio 116; el GF presentó 51,3% de pérdidas. Los escolares formados por el GM obtuvieron puntuaciones medianas significativamente mayores tanto en conocimiento teórico (6,7; RIC=1,8 vs 4,7; RIC=3,1; p<0,001) como en todas las habilidades prácticas a excepción de *reconocer un situación de emergencia*. En el GF, la enseñanza sobre RCP con un cuento y un vídeo de dibujos animados logró puntuaciones significativamente mejores en cinco conocimientos y en cuatro habilidades que con solo un cuento.

**Conclusiones:**

La implementación de recursos educativos no tecnológicos, cómo cuentos y dibujos animados, en la enseñanza de la RCP en escolares de primaria aumenta los conocimientos y habilidades. Los escolares formados por los maestros en el ámbito educativo han aprendido significativamente más que los formados por la familia y, dentro del ámbito familiar, la enseñanza sobre RCP fue más eficaz mediante un cuento y un vídeo de dibujos animados que cuando simplemente disponen del cuento.

## Abstract (en)

**Background:**

We compared the outcome of training schoolchildren how to perform CPR by parents/legal guardians in the family environment versus by teachers at school.

**Methods:**

Randomized study of CPR learning in primary school children (1st and 2nd grades) in the Region of Murcia. Parents/legal guardians (family group) and teachers (teacher group) trained the children using didactic material adapted for that age population (one story and one cartoon video) from the educational series *Jacinto y sus Amigos©*. We evaluated eight theoretical knowledge questions and five practical skills.

**Results:**

One hundred and sixty schoolchildren were selected and 116 completed the study; in the family group, 51.3% did not finish the study. Children trained by teachers obtained significantly higher median scores in comparison with the family group both in theoretical knowledge (6.7; IQR=1.8 vs 4.7; IQR=3.1, respectively; p < 0.001) and in all practical skills except for “recognizing an emergency situation”. Children in the family group, learning CPR with one story and one cartoon video achieved significantly better scores in five theoretical knowledge questions and four skills than with only the story.

**Conclusions:**

Using non-technological educational resources, e.g., stories and animated cartoons for teaching CPR to primary school students, increases their knowledge and skills. Schoolchildren trained in the educational environment acquired more knowledge and skills than those trained by parents. Within the family environment, CPR teaching was more effective through one story and one cartoon video than when only the story was used.

## INTRODUCCIÓN

En los países occidentales, la parada cardiorrespiratoria (PCR) es la principal causa de muerte prematura. Prevalece la etiología cardiaca, ocasionando alrededor de 15.000 a 25.000 muertes al año. La enfermedad cardiovascular, mata 65 veces más que los accidentes de tráfico^1^^,^^2^. Como solución a esta situación, están las maniobras de reanimación cardiopulmonar (RCP), estandarizadas y aceptadas internacionalmente, intentando suplir y rehabilitar la respiración y circulación espontánea^3^. La mejoría en la supervivencia y disminuir las secuelas potenciales dependerá de la cadena de supervivencia, que está formada por cuatro eslabones vitales para la resucitación^4^: reconocimiento precoz de parada y pedir ayuda al 112, RCP precoz, desfibrilación precoz y, por último, el soporte vital avanzado.

La posibilidad de sobrevivir ante una PCR extrahospitalaria puede aumentar hasta cinco veces, si los primeros intervinientes comienzan las maniobras de RCP lo antes posible^3^. Dado que la mayor parte de las PCR se dan en el medio extrahospitalario, y la mayoría se producen en el ámbito doméstico, el conocimiento que tengan los testigos sobre primeros auxilios y maniobras de RCP juegan un papel fundamental para que la intervención sea lo más exitosa posible^4^^-^^6^. Por eso es necesario que no solo el personal sanitario, bomberos, policías, socorristas, etc., sepan y conozcan cómo actuar, sino que es necesaria la educación para la salud en maniobras de RCP básica para la población. En nuestro país, solo el 12% de la población reconoce saber las técnicas sobre RCP por lo que si se formará al 20% de la población salvaríamos 100.000 vidas anuales^2^^,^^7^.

En el año 2015 la declaración *Kids Save Lives* (Los Niños Salvan Vidas) fue aprobada por la Organización Mundial de la Salud (OMS)^8^. Esta declaración ha puesto de manifiesto lo importante que resulta la enseñanza de Reanimación Cardiopulmonar a todos los niños del mundo. La escuela debe proveer a los estudiantes para cumplir con los diferentes desafíos del mundo moderno, incluida la asistencia a las víctimas en respuesta a emergencias, la atención de su propia seguridad y la seguridad de las víctimas y los testigos. Numerosos estudios han concluido que los niños pueden aprender primeros auxilios y llevarlos a cabo de forma segura^7^^-^^10^. Las recomendaciones del Consejo Europeo de Reanimación de 2021 aconsejan enseñar primeros auxilios en todos los niveles de la educación^3^. En los países en los que la formación en reanimación está integrada en los programas educativos se ha observado un aumento de las tasas de supervivencia^1^^,^^2^.

La iniciativa *Kids Save Lives*^8^ afirma que mediante la introducción de solo dos horas de enseñanza de RCP al año, para todos los niños mayores de 12 años, es suficiente para aprender a realizar una RCP, mejorando con ello la salud de la población mundial si todos los colegios con educación secundaria implantaran su formación. Históricamente se han utilizado diversas metodologías^10^ como: *kits* de auto-instrucción, el aprendizaje basado en medios informáticos, y el aprendizaje mediante formación realizada por profesores o personal sanitario invitado al centro escolar. Los niños disfrutan de la formación y la mayoría pueden compartir sus conocimientos con familiares y amigos^9^. En el año 2021, el Consejo Europeo de Resucitación (ERC) recomendó que el aprendizaje debería ser progresivo, introduciendo la formación en primeros auxilios y RCP desde la educación primaria hasta alcanzar los conceptos más complejos en la educación secundaria^11^. Los contenidos por debajo de los 12 años deberían centrarse en que los niños aprendan el trinomio *comprueba, llama y comprime*^3^.

La mayor parte de los estudios consultados destacan dos problemas principales: la escasez de material pedagógico en RCP adaptado a estas edades y la falta de integración de la familia como un posible agente formador de los niños.

Por todo lo expuesto, el objetivo principal fue analizar los resultados obtenidos en la formación de RCP, comparando a niños formados por los padres en el ambiente familiar con otros formados por los maestros en el ambiente educativo. Como objetivo secundario se planteó la eficacia del uso de un cuento y un vídeo de dibujos animados para la enseñanza de RCP.

## MÉTODOS

Estudio aleatorizado del aprendizaje de RCP en escolares de Educación Primaria de la Región de Murcia, comparando los resultados obtenidos cuando los formadores son los maestros o los progenitores. La formación consistió en la lectura del cuento de formación en RCP de *Jacinto y sus Amigos*© (http://www.jacintoysusamigos.com), tres días durante una semana (lunes, miércoles y viernes), y en la visualización de un vídeo de dibujos animados (https://youtu.be/sya_zuYfqOY), dos días en la misma semana (martes y jueves).

Este estudio fue aprobado por el Comité de Ética de la Universidad Católica de Murcia (UCAM). Se obtuvo el consentimiento por parte de la dirección del centro escolar, y el consentimiento personal firmado por parte de los progenitores y/o tutores legales de cada escolar participante.

### Selección de la muestra

La población de estudio es el alumnado de 1° y 2° de Educación Primaria. La muestra de este estudio se obtuvo mediante convocatoria abierta a través del registro de la Conserjería de Educación (Región de Murcia) hasta alcanzar la población de estudio deseada. Los centros seleccionados fueron aleatorizados (muestreo aleatorio simple) en Grupo Familia (GF) y Grupo Maestros (GM), según si el alumnado iba a ser formado por padres o por maestros. Independientemente del grupo, a cada escolar participante se le entregó un cuento y el enlace a un vídeo de animación.

El cálculo del tamaño muestral se realizó según un incremento medio esperado 2,5 puntos en la nota teórica tras la intervención, con desviación típica 1 punto, un 90% de potencia para detectar el incremento medio esperado y un error de tipo I del 5%. Los incrementos esperados los hemos obtenido de un estudio similar^12^. Se estimó necesario contar con 65 escolares en cada uno de los dos grupos de este estudio. Contando con una tasa de abandono del 20% (13 escolares/grupo), se determinó la necesidad de 156 participantes; finalmente se optó por incluir una muestra de estudio de 160 escolares en total.

### Evaluación de conocimientos de RCP

Puesto que no existen cuestionarios teóricos validados para estas edades, se creó un cuestionario *ad hoc* (Anexo I) consistente en la realización de ocho actividades con una puntuación total de 10 puntos. El conocimiento teórico (CT) se valoró otorgando un punto a cada actividad (1- llamada al 112, 2- reconocer el desfibrilador externo automático (DEA), 3- relación compresiones / ventilaciones, 4- lugar de compresión, 6- posición lateral de seguridad, 8- conducta PAS (proteger, avisar y socorrer), excepto a las actividades número 5 (uso del DEA) y 7 (actuación PCR), a las que se otorgaron 2 puntos por ser más complejas. A mayor puntuación, mayor CT.

Las habilidades prácticas (HP) se evaluaron mediante cinco actividades: 1) reconocer una situación de emergencia, 2) llamar al 112, 3) localizar el punto de las compresiones torácicas, 4) colocación de los brazos para hacer las compresiones, y 5) reconocer un DEA y sus partes básicas (parches, botón de encendido, etc.) (Anexo II). En la evaluación de las cinco habilidades prácticas se puntuó con 1 punto si no se realizaba la actividad, con 2 puntos si se realizaba parcialmente y con 3 puntos si se realizaba correctamente. A mayor puntuación, mayores HP. Todas las valoraciones fueron consensuadas por dos instructoras en Soporte Vital Básico (SVB) y DEA (las autoras CCM y LJR).

### Análisis estadístico

La variable principal del estudio fue la mediana de CT y la variable secundaria la mediana de HP. Una vez recogidos todos los datos mediante el programa Microsoft Excel®, estos se analizaron mediante el programa SPSS v.21®. El análisis se realizó mediante comprobación de la distribución normal (test Kolmogorov Smirnov) y cálculo de mediana y rango intercuartílico (RIC) si no seguían normalidad. La comparación de resultados se realizó por medio de la prueba U de Mann-Withney. Las diferencias se consideraron estadísticamente significativas si p<0,05.

## RESULTADOS

**Figura 1:**
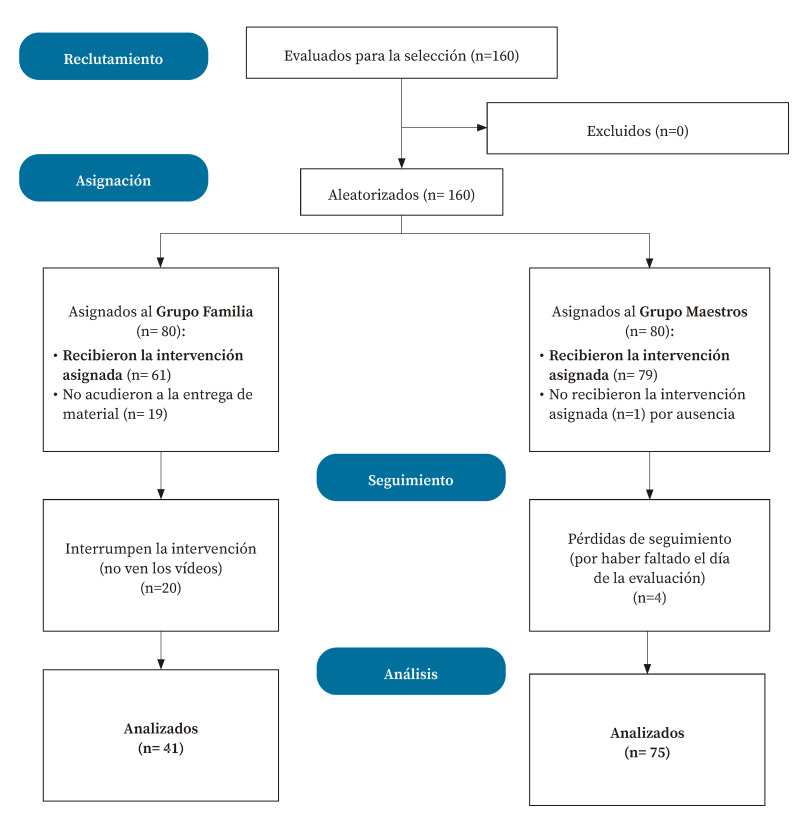
Diagrama de flujo de los participantes (basado en el esquema Consort).

De los 160 escolares aleatorizados en dos grupos, 116 compusieron la muestra final del estudio (Figura 1). En el GF se dio pérdida de muestra por no acudir a la entrega del material (n=19; 23,8%) y, ya durante el seguimiento, por no cumplir con la visualización de los vídeos (n=20; 25%), tarea que era responsabilidad de los progenitores. Debido a esta incidencia, se decidió crear dos subgrupos *a posteriori* en el GF, a fin de comparar el aprendizaje con vídeo y un cuento (GF-VC, n=41) y el aprendizaje solo con un cuento (GF-C, n=20).

### Conocimientos teóricos adquiridos

Los escolares formados por los maestros obtuvieron mayor mediana de CT que los formados por los familiares (6,7; RIC=1,8 vs 4,7; RIC=3,1; p<0,001) (Tabla 1). Las medianas de puntuación de CT obtenidas por los dos subgrupos del GF indican que los escolares que visualizaron el cuento y el vídeo mostraron más CT que los que solamente vieron el cuento (5,9; RIC=2,8 vs 3,3; RIC=1,5; p<0,001). Estos resultados ponen de manifiesto que los niños obtuvieron más conocimientos cuando ven los vídeos y un cuento que simplemente con el cuento.

**Tabla 1:** Comparación de conocimientos teóricos según grupo y subgrupo familiar

Conocimiento teórico	Grupo		p*	Grupo Familia		p*
	Familia	Maestros		VC	C	
	(n=61)	(n=75)		(n=41)	(n=20)	
	Mediana (RIC)	Mediana (RIC)		Mediana (RIC)	Mediana (RIC)	
1. Llamada al 112	1,0 (0)	1,0 (0)	0,118	1,0 (0)	1,0 (0)	0,618
2. Reconocer el DEA	1,0 (0)	1,0 (0)	0,507	1,0 (0)	1,0 (0)	0,067
3. Relación compresiones / ventilaciones	0 (1,0)	1,0 (0)	<0,001	1,0 (1,0)	0 (0)	<0,001
4. Lugar de compresiones	1,0 (1,0)	1,0 (1,0)	0,455	1,0 (1,0)	0 (1,0)	0,02
5. Uso del DEA#	0 (1,0)	0,5 (0,5)	<0,001	0,5 (1,0)	0 (0,12)	0,004
6. Posición lateral de seguridad	0 (0,5)	1 (0,5)	<0,001	0,5 (0,75)	0 (0,06)	0,001
7. Actuación PCR#	0,4 (0,5)	0,4 (0,6)	0,104	0,4 (0,8)	0,4 (0,4)	0,21
8. Conducta PAS	0,66 (0,67)	1 (0)	<0,001	1,0 (0,67)	0,49 (0,66)	0,018

RIC: rango intercuartílico; *: U de Mann-Whitney; #: valorada con 2 puntos; DEA: desfibrilador externo automático; PCR: parada cardio-respiratória; PAS: proteger-avisar-socorrer. Resultados estadísticamente significativos, en negrita.

### Habilidades Prácticas

Las medianas de puntuación de HP fueron significativamente mejores para el GM que para el GF en todas las habilidades excepto para la habilidad 1 (reconocer una situación de emergencia, que todos los escolares realizaron correctamente). Dentro del GF, se observaron diferencias estadísticamente significativas entre los subgrupos familiares que vieron y no vieron los vídeos: las puntuaciones de HP del GF-C fueron peores que las del GF-VC excepto la habilidad 1 (Tabla 2).

**Tabla 2:** Comparación de habilidades prácticas según grupo y subgrupo familiar.

Habilidades prácticas	Grupo		p*	Grupo Familia		p*
	Familia	Maestros		VC	C	
	(n=61)	(n=75)		(n=41)	(n=20)	
	Mediana (RIC)	Mediana (RIC)		Mediana (RIC)	Mediana (RIC)	
1. Reconocer una situación de emergencia	3,0 (0,0)	3,0 (0,0)	0,273	3,0 (0,0)	3,0 (0,0)	0,162
2. Llamar al 112	2,0 (1,0)	2,0 (1,0)	0,002	2,0 (2,0)	1,0 (0,25)	<0,001
3. Localizar el punto de las compresiones torácicas	3,0 (0,0)	3,0 (0,0)	0,004	3,0 (0,0)	3,0 (1,0)	<0,001
4. Colocación de los brazos para hacer las compresiones	3,0 (2,0)	3,0 (0,0)	<0,001	3,0 (1,0)	1,0 (1,25)	0,001
5. Reconocer un DEA y sus partes básicas	2,0 (1,0)	3,0 (1,0)	<0,001	2,0 (1,0)	1,0 (1,0)	<0,001

RIC: rango intercuartílico; *: U de Mann-Whitney; DEA: desfibrilador externo automático. Resultados estadísticamente significativos, en negrita.

## DISCUSIÓN

Los resultados obtenidos indican que los escolares formados mediante un cuento y un video de dibujos animados logran mejorar sus conocimientos de RCP, como ya había descrito el gobierno escocés^13^, si bien con incrementos algo inferiores a los obtenidos en el presente estudio.

Por otro lado, si tenemos en cuenta el ámbito donde se ha realizado la formación, observamos que en el ámbito escolar se obtuvieron mejores resultados que en el ámbito familiar, tanto en conocimientos como en habilidades, dado que los profesores están capacitados para formar en RCP^14^^-^^17^. Un estudio longitudinal de seis años de seguimiento mostró que los escolares retuvieron la formación sobre RCP incluso hasta tres años tras la formación^16^. Además, no encontraron diferencias en los conocimientos adquiridos, dependiendo de los distintos grupos de instructores (profesionales de salud y sus profesores)^16^. Como formadores, el profesorado ofrece varias ventajas: dispone de las habilidades educativas necesarias, actúa como modelo para sus alumnos, la formación puede organizarse más fácilmente y sería menos costosa. Los materias escolares de Biología y de Educación Física podrían ser las más adecuadas para ofrecer oportunidades para el entrenamiento de la RCP en la escuela^15^^,^^18^^-^^20^. La menor adquisición de conocimientos y habilidades en el ámbito familiar concuerda con Talavera y col^21^, quienes determinaron dificultades y limitaciones como falta de tiempo por parte de los progenitores debido a su alta carga de trabajo o escasa colaboración de las familias.

La mayor adquisición de conocimientos y habilidades añadiendo un vídeo al cuento pone de manifiesto la importancia de utilizar métodos que combinen diversos recursos educativos, y es congruente con los resultados de Nord y col, donde los conocimientos sobre RCP fueron mejores si se añadía un vídeo de 50 minutos^18^, tiempo similar al empleado en nuestro estudio. Aunque nuestros resultados son estadísticamente significativos, cabe destacar que la creación de grupos de análisis a *posteriori* es una limitación metodológica; además, existen diferencias importantes en el tamaño de cada uno de los dos subgrupos.

Existe mucha controversia sobre el momento en el que los escolares son capaces de realizar compresiones torácicas de calidad. Los contenidos por debajo de los 12 años deberían centrarse en que los niños aprendan el trinomio *comprueba, llama y comprime*^3^, aunque estudios más recientes han demostrado que con un peso medio de 40 kilos, ya tienen las condiciones físicas para llevar a cabo correctamente las técnicas de RCP^9^^,^^14^^,^^22^. La muestra incluida en nuestro estudio tenía una edad y un peso inferior, por lo que se decidió no determinar la profundidad y el ritmo en la compresiones y simplemente si sabían y eran capaces de demostrar la habilidad. Este hecho debe tenerse en consideración cómo un posible sesgo y limitación del estudio.

El ERC recomienda involucrar a las familias y al entorno del niño en el aprendizaje de la RCP^3^. En la literatura científica se apunta que quien debe hacerse cargo de la educación para la salud sean las familias junto con el personal sanitario^23^^,^^24^. Pero también podría pensarse en que la responsabilidad sea del profesorado. Louis y col señalan que el primer paso es formar a los maestros para que estos se sientan seguros^25^. Los profesionales sanitarios pueden hacer *píldoras formativas* esporádicas en los centros escolares para reforzar estos conocimientos. En nuestro estudio percibimos una actitud muy colaborativa por parte del profesorado, a pesar de que no se realizó una formación específica para estos colectivos, simplemente se les ofreció los recursos didácticos (cuento y vídeo).

Muchas de las iniciativas actuales se centran en el uso de la tecnología cómo vídeos^22^, videojuegos formativos^26^ o realidad virtual^27^. Mediante este trabajo hemos podido demostrar que el uso de recursos no tecnológicos puede ser igualmente eficiente.

Las principal limitación de este trabajo es que debido a la ausencia de cuestionarios validados para valorar conocimientos teóricos y habilidades prácticas en RCP adaptadas a esta edad, en este estudio hemos usado un cuestionario *ad hoc*. Otra limitación es la pérdida de seguimiento de participantes del GC, aunque *a priori*, todos accedieron a participar al inicio del estudio.

La enseñanza de la RCP a los escolares y adolescentes debe hacerse de manera holística incluyendo los ámbitos escolar y familiar^3^^,^^12^^,^^23^. La estrategia de otros programas educativos similares ha sido comenzar a formar a la población desde las escuelas para que en unos años toda la sociedad tenga los conocimientos necesarios. El trabajo en equipo es fundamental, por lo que la transmisión de conocimientos de RCP debe hacerse de manera coordinada entre sanitarios, maestros y familiares. La implicación de maestros y familiares en esta área hace que de manera indirecta ellos también se formen. Por último, que el escolar vea que todo su entorno está motivado y preocupado por su formación en RCP, pensamos que genera un efecto sinérgico positivo al incluir a las personas adultas que suelen ser sus referentes.

La principal conclusión de este trabajo es que la implementación de recursos educativos no tecnológicos para la enseñanza de la RCP en escolares de educación primaria (hasta 12 años) aumenta los conocimientos y habilidades. Los escolares formados por los maestros en el ámbito educativo han aprendido significativamente más que los formados por la familia y, dentro del ámbito familiar, la enseñanza sobre RCP fue más eficaz mediante un cuento y un vídeo de dibujos animados que cuando simplemente disponen del cuento.

## Figures and Tables

**Figure:**
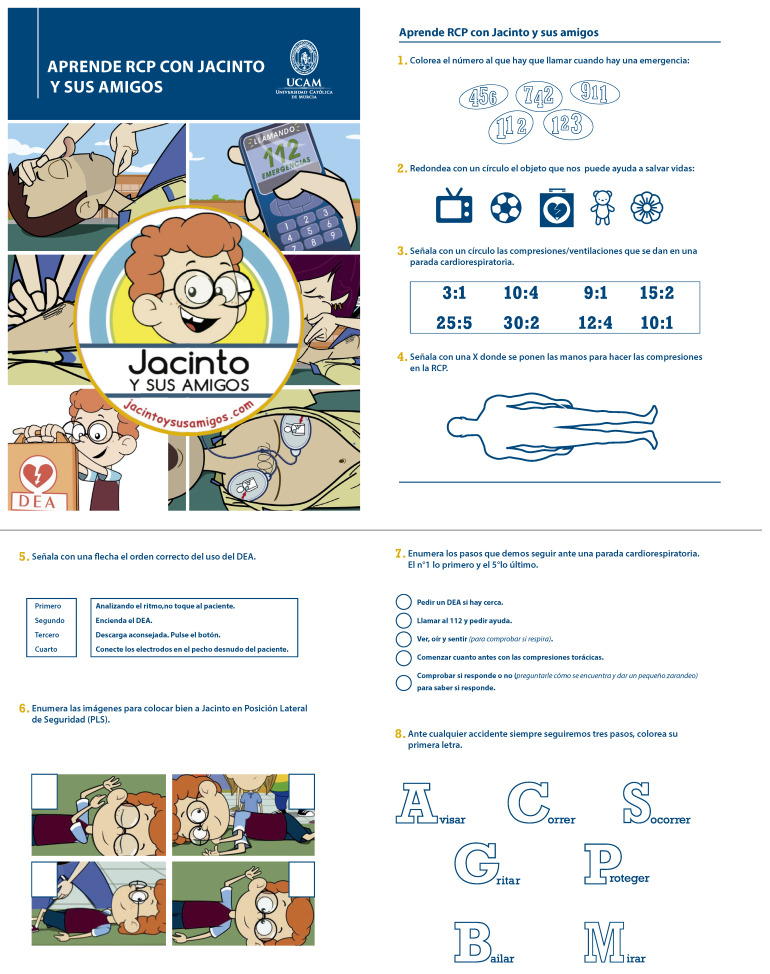
Cuestionario elaborado para la evaluación teórica de conocimientos*

* Reproducido con permiso y autorización de los autores

**Figure:**
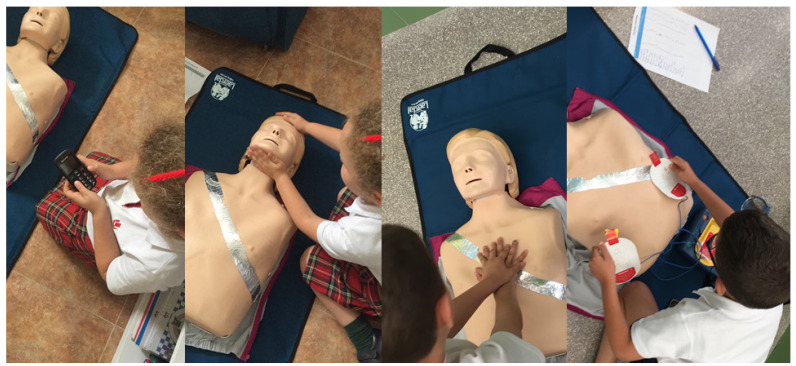
Imágenes de la evaluación práctica de habilidades

## References

[1] Rosell Ortiz F, López Messa JB, Mellado Vergel FJ (2012). Registro español de parada cardiaca extrahospitalaria. Presentación del Proyecto de Investigación 'Aspectos epidemiológicos, variabilidad y supervivencia en la atención a la parada cardiaca extrahospitalaria por servicios de emergencia en España'. REMI.

[2] Palacio Villazón R, Nonide Robles M, Carreño Morán F, López Roldan L, Cao Fernández A (2015). Proyecto "con tus manos puedes salvar vidas". RqR Enfermería Comunitaria (Revista de SEAPA).

[3] Perkins GD, Graesner JT, Semeraro F, Olasveengen T, Soar J, Lott C (2021). European Resuscitation Council Guidelines 2021: Executive summary. Resuscitation.

[4] Roman-Patrik L, Van Aken H, Mölhoff T, Weber T, Rammert M, Wild E (2016). Kids save lives: a six-year longitudinal study of schoolchildren learning cardiopulmonary resuscitation: Who should do the teaching and will the effects last?. Resuscitation.

[5] Consejo Español de Resucitación Cardiopulmonar http://www.cercp.org/el-cercp/consejo-espanol-de-rcp.

[6] Folke F, Gislason GH, Lippert FK, Nielsen SL, Weeke P, Hansen ML (2010). Differences between out-of-hospital cardiac arrest in residential and public locations and implications for public-access defibrillation. Circulation.

[7] García Vega FJ, Montero Pérez FJ, Encinas Puente RM (2008). La comunidad escolar como objetivo de la formación en resucitación: la RCP en las escuelas. Emergencias.

[8] Böttiger BW, Van Aken H (2015). Kids save lives. Resuscitation.

[9] Miro O, Díaz N, Sánchez M (2017). Aprender reanimación cardiopulmonar desde la escuela. Emergencias.

[10] Fradejas Sastre V, Pérez Velasco P (2013). Importancia de una comunidad educativa formada en técnicas de reanimación cardiopulmonar. Nuberus Científica.

[11] Sociedad Española de Cardiología (2015). La enfermedad cardiovascular mata en España 65 veces más que los accidentes de tráfico.

[12] Cerezo Espinosa C, Nieto Caballero S, Juguera Rodríguez L, Castejón-Mochón JF, Segura Melgarejo F, Sánchez Martínez CM (2018). Learning cardiopulmonary resuscitation theory with face-to-face versus audiovisual instruction for secondary school students: a randomized controlled trial. Emergencias.

[13] The Scottish Government (2016). Out-of-Hospital Cardiac Arrest - A Strategy for Scotland Review 2015-16.

[14] Navarro Patton R, García Marín P, Rodríguez Fernández JE (2015). Conocimientos previos y adquiridos tras una jornada de formación sobre primeros auxilios en futuros docentes de Educación Física. Sportis.

[15] Böttiger BW, Bossaert LL, Castrén M, Cimpoesu D, Georgiou M, Greif R, Board of European Resuscitation Council (ERC) (2016). Kids Save Lives -ERC position statement on school children education in CPR. "Hands that help - Training children is training for life". Resuscitation.

[16] Bohn A, Lukas RP, Breckwoldtc J, Böttiger BW, Van Aken H (2015). 'Kids save lives': why schoolchildren should train in cardiopulmonary resuscitation. Curr Opin Crit Care.

[17] Hori S, Suzuki M, Yamazaki M, Aikawa N, Yamazaki H (2016). Cardiopulmonary resuscitation training in schools: a comparison of trainee satisfaction among different age groups. Keio J Med.

[B18] Nord A, Svensson L, Hult H, Kreitz-Sandberg S, Nilsson L (2016). Effect of mobile application-based versus DVD-based CPR training on students' practical CPR skills and willingness to act: a cluster randomised study. BMJ Open.

[19] Lukas RP, Akena HV, Moldoff T, Weber T, Rammert M, Wild E (2016). Kids save lives: a six-year longitudinal study of schoolchildren learning cardiopulmonary resuscitation: Who should do the teaching and will the effects last?. Resuscitation.

[20] Mpotos N, Vekeman E, Monsieurs K, Derese A, Valcke M (2014). Knowledge and willingness to teach cardiopulmonary resuscitation: a survey amongst 4273 teachers. Resuscitation.

[21] Talavera Ortega M, Gavidia Catalán V (2007). Dificultades para el desarrollo de la educación para la Salud en la escuela. Opiniones del profesorado. Didáctica de las Ciencias Experimentales y Sociales.

[22] Zinckernagel L, Malta Hansen C, Rod MH, Folke F, Torp-Pedersen C, Tjørnhøj-Thomsen T (2016). What are the barriers to implementation of cardiopulmonary resuscitation training in secondary schools? A qualitative study. BMJ Open.

[23] Cave DM, Aufderheide TP, Beeson J, Ellison A, Gregory A, Hazinski MF (2011). Importance and implementation of training in cardiopulmonary resuscitation and automated external defibrillation in schools: A science advisory from the American Heart Association. Circulation.

[24] Greif R, Lockey A, Breckwoldt J, Carmona F, Conaghan P, Kuzovlev A (2021). European Resuscitation Council Guidelines 2021: Education for resuscitation. Resuscitation.

[25] Louis CJ, Beaumont C, Velilla N, Greif R, Fernandez J, Reyero D (2022). The "ABC SAVES LIVES": A schoolteacher-led basic life support program in Navarra, Spain. SAGE Open.

[26] López MM, Ortega SA, Ríos MP, Ruiz RM (2022). Serious Games for Health, una herramienta efectiva para el entrenamiento de los profesionales sanitarios. Aten Primaria.

[27] Alcázar Artero PM, Pardo Rios M, Greif R, Ocampo Cervantes AB, Gijón-Nogueron G, Barcala-Furelos R (2023). Efficiency of virtual reality for cardiopulmonary resuscitation training of adult laypersons: A systematic review. Medicine (Baltimore).

